# Advancement in Anaerobic Ammonia Oxidation Technologies for Industrial Wastewater Treatment and Resource Recovery: A Comprehensive Review and Perspectives

**DOI:** 10.3390/bioengineering12040330

**Published:** 2025-03-22

**Authors:** Pradeep Singh, Monish Bisen, Sourabh Kulshreshtha, Lokender Kumar, Shubham R. Choudhury, Mayur J. Nath, Manabendra Mandal, Aman Kumar, Sanjay K. S. Patel

**Affiliations:** 1 School of Biotechnology, Faculty of Applied Sciences and Biotechnology, Shoolini University, Solan 173229, Himachal Pradesh, India; crepipradeep8888@gmail.com (P.S.); monishbisen@shooliniuniversity.com (M.B.); sourabhkulshreshtha@shooliniuniversity.com (S.K.); 2 Cancer Biology Laboratory, Raj Khosla Centre for Cancer Research, Shoolini University, Solan 173229, Himachal Pradesh, India; 3 Department of Molecular Biology and Biotechnology, Tezpur University, Napaam, Tezpur 784028, Assam, India; royshubham.15apr@gmail.com (S.R.C.); mjntez2016@gmail.com (M.J.N.); mandal@tezu.ernet.in (M.M.); 4 Department of Biotechnology, Hemvati Nandan Bahuguna Garhwal University (A Central University), Srinagar 246174, Uttarakhand, India; amankumar94266@gmail.com

**Keywords:** anaerobic ammonia oxidation technologies, industrial wastewater, membranes, water pollution, wastewater treatment technologies

## Abstract

Anaerobic ammonium oxidation (anammox) technologies have attracted substantial interest due to their advantages over traditional biological nitrogen removal processes, including high efficiency and low energy demand. Currently, multiple side-stream applications of the anammox coupling process have been developed, including one-stage, two-stage, and three-stage systems such as completely autotrophic nitrogen removal over nitrite, denitrifying ammonium oxidation, simultaneous nitrogen and phosphorus removal, partial denitrification-anammox, and partial nitrification and integrated fermentation denitritation. The one-stage system includes completely autotrophic nitrogen removal over nitrite, oxygen-limited autotrophic nitrification/denitrification, aerobic de-ammonification, single-stage nitrogen removal using anammox, and partial nitritation. Two-stage systems, such as the single reactor system for high-activity ammonium removal over nitrite, integrated fixed-film activated sludge, and simultaneous nitrogen and phosphorus removal, have also been developed. Three-stage systems comprise partial nitrification anammox, partial denitrification anammox, simultaneous ammonium oxidation denitrification, and partial nitrification and integrated fermentation denitritation. The performance of these systems is highly dependent on interactions between functional microbial communities, physiochemical parameters, and environmental factors. Mainstream applications are not well developed and require further research and development. Mainstream applications demand a high carbon/nitrogen ratio to maintain levels of nitrite-oxidizing bacteria, high concentrations of ammonium and nitrite in wastewater, and retention of anammox bacteria biomass. To summarize various aspects of the anammox processes, this review provides information regarding the microbial diversity of different genera of anammox bacteria and the engineering aspects of various side streams and mainstream anammox processes for wastewater treatment. Additionally, this review offers detailed insights into the challenges related to anammox technology and delivers solutions for future sustainable research.

## 1. Introduction

**Figure 1:**
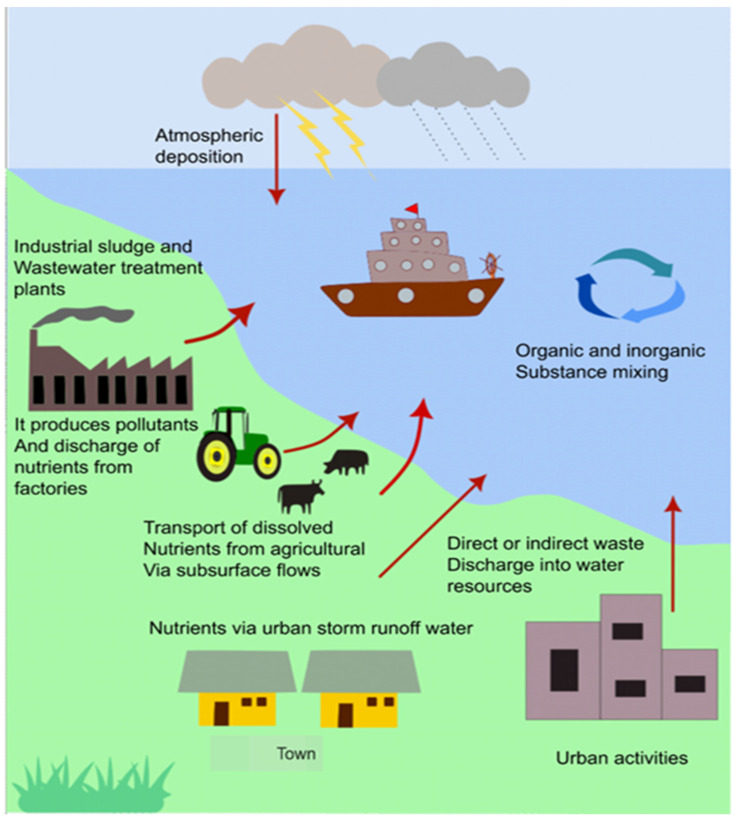
Pollute discharges from multiple sources contaminate groundwater.

Industrial wastewater is a global ecological concern due to high nitrogen-containing contaminants such as ammonia (NH_3_), nitrite, and nitrates [1,2]. Among these contaminants, ammonia is most frequently discharged into aquatic ecosystems, thereby critically affecting the ecological equilibrium [3,4,5]. Ammonia, a colorless and odorless compound, is a poisonous gas in its compressed anhydrous form [3]. Given the potential risks associated with nitrogen-based discharges, the US Environmental Protection Agency has updated the criteria for water quality for nitrogen-based discharges. According to a report by the World Health Organization (2017), ammonia should be less than 0.2 mg/L in surface or groundwater. The increase in the ammonium concentration was due to effluent discharge from municipal, industrial, and agricultural wastewater [6,7]. Further, industries, including textile, processed food plants, fertilizer industries, and rubber-producing plants, release high concentrations of ammonia into the water ecosystem, as shown in Figure 1 [1,2], resulting in eutrophication, characterized by excessive growth of algae and cyanobacteria. An increase in ammonium concentration reduces dissolved oxygen (DO), producing cyanotoxins that harm aquatic life [8]. Ammonia also interferes with oxygen transfer in the gills of fishes, causing death [2]. Furthermore, polluted water and the consumption of contaminated fish adversely affect human health [1,9]. Therefore, the elimination of nitrogen-containing contaminants is crucial (Figure 1).

Researchers have been prompted by the European Environment Agency and other indigenous ecological agencies to develop ecological and cost-effective methods for removing ammonium contaminants from industrial effluents. Several techniques have been developed for eliminating these contaminants [10,11,12]. Membrane filtering techniques comprise physiochemical processes such as microfiltration, nanofiltration, ultrafiltration, and reverse osmosis [13]. Ion exchange via chemical precipitation [14] and other techniques such as photocatalytic oxidation, aeration, and air stripping have also been used [1,15]. However, the implementation of these techniques faces challenges, such as high operating expenses, skilled manpower, and secondary waste production [16,17]. Traditional biological approaches for nitrogen elimination processes, for example, denitrification and nitrification, have been successfully employed to remediate nitrogen contaminants in both domestic sewage and industrial wastewater [18,19,20,21].

The two-step nitrification process is catalyzed by obligate chemolithoautotrophic prokaryotic organisms such as ammonia-oxidizing archaea (AOA), ammonia-oxidizing bacteria (AOB), and nitrite-oxidizing bacteria (NOB) [1]. Finally, facultative anaerobic heterotrophic bacteria catalyze an anaerobic process known as denitrification [22]. Ammonium (NH_4_^+^) oxidation by AOB produces nitrite (NO_2_^−^); NOB convert NO_2_^−^ into nitrate (NO_3_^−^); and de-nitrifiers reduce NO_3_^−^ to dinitrogen gas (N_2_) [23]. However, these traditional approaches have limitations, such as high amounts of external carbon sources, slow reaction rates, unnecessary sludge generation, greenhouse gas emissions, and high energy consumption and costs [23,24]. Therefore, these approaches require urgent innovation. The use of anaerobic ammonium oxidation (anammox) has drawn attention as an efficient and sustainable method for treating industrial discharge with high ammonia content [25,26].

The 6 identified genera with more than 21 species of autotrophic anammox bacteria are Anammoximicrobium moscowii, Anammoxoglobus propionicus, Brocadia anammoxidans, Jettenia asiatica, Kuenenia stuttgartiensis, and Scalindua wagneri [27]. Interestingly, these bacteria are mostly sampled from laboratory-scale reactors or industrial wastewater treatment facilities [16,28]. The global issue of industrial wastewater, particularly contaminated with nitrogen-containing contaminants such as ammonia, poses substantial risks to ecological equilibrium and human wellbeing. This review examines the diverse methodologies employed to eliminate ammonium contaminants, encompassing physiochemical processes, ion exchange, and traditional biological techniques such as nitrification and de-nitrification. Among these approaches, anammox has emerged as a promising and sustainable strategy for effectively treating industrial wastewater with high ammonia concentrations. By highlighting the significance of industrial wastewater treatment, the limitations inherent in the current methods, and the potential of anammox bacteria, this study contributes to understanding ammonia content in industrial wastewater via innovative and environmentally friendly approaches.

## 2. Anammox

Anammox refers to the biotic method of substituting nitrogen with dinitrogen gas in an anoxic environment in which nitrite and ammonium act as oxidants and reductants, respectively [29,30,31]. Theoretical predictions of the anammox process were initially made using nitrogen profiles and thermodynamic calculations [32]. Anammox is essential for transforming half the nitrogen in marine ecosystems under varying salinity and temperature conditions [33]. This was attributed to the bacteria’s high salt tolerance, temperature resistance, and rate of organic waste depletion. Anammox has emerged as a highly effective method for treating industrial wastewater and offers several distinct advantages over conventional techniques. This process can lower organic carbon requirements by 100%, reduce oxygen demand by 60%, and decrease sludge production by 90% [34]. Consequently, research on industrial discharge treatment has heavily emphasized anammox processes. The discovery of anammox has considerably improved our understanding of the overall nitrogen cycle. This knowledge will pave the way for developing novel biological nitrogen elimination techniques for wastewater treatment [35]. Through continued research and innovation in this field, we can further optimize the use of anammox in industrial wastewater treatment while also gaining a deeper understanding of its role in shaping the nitrogen cycle.

**Figure 2:**
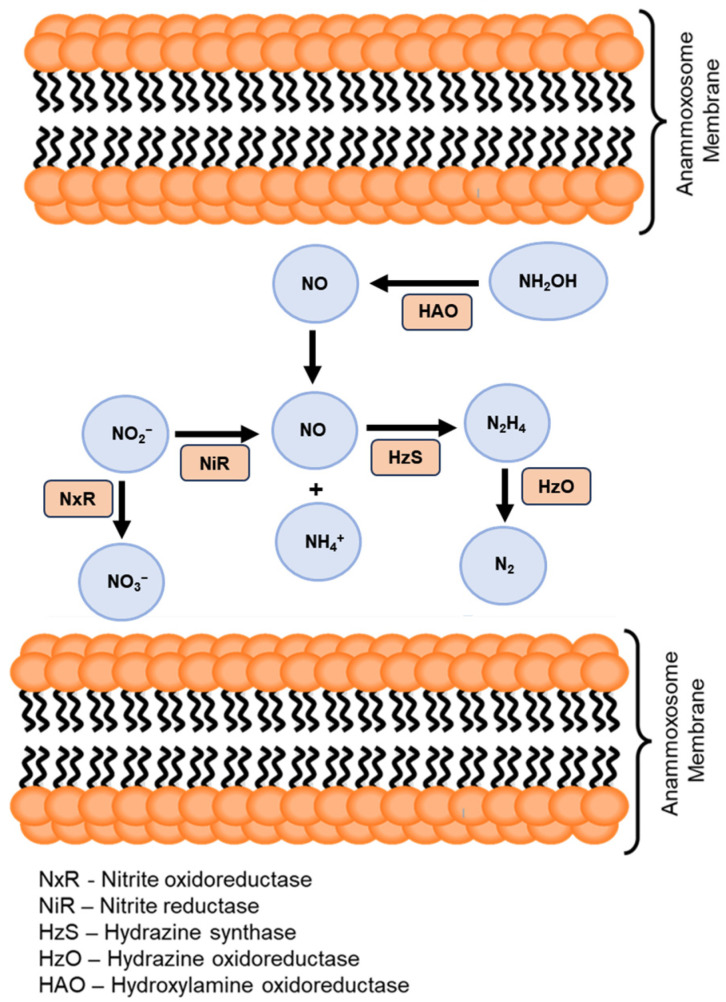
Chemical molecules and enzymes involved in the anammox process.

**Figure 3:**
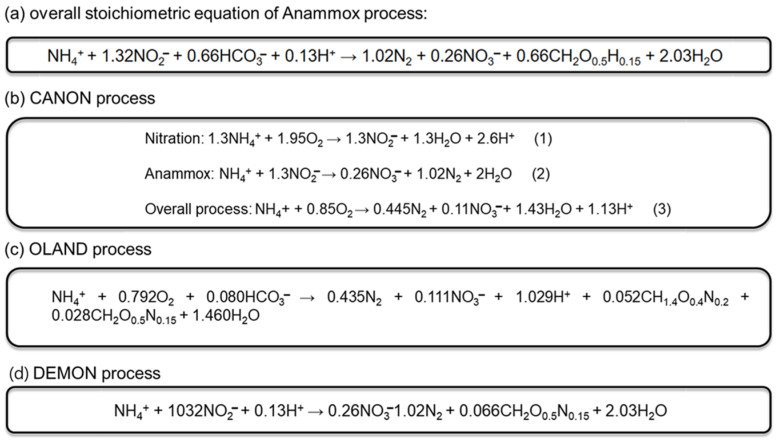
Stoichiometric equations of various processes of anammox technology used for industrial wastewater treatment: (**a**) overall stoichiometric equation of anammox process, (**b**) CANON process, (**c**) OLAND process, (**d**) DEMON process.

The first known anammox bacteria were isolated using density gradient centrifugation [36]. This study revealed several unique phenotypic characteristics distinct from those of this class of bacteria. For instance, the cells were observed to have a red-colored, crateriform, bowl-shaped structure on the surface. Subsequent studies corroborated these observations [3,36]. Additionally, an intracellular compartment called the anammoxosome was identified along with ladderane lipids in the intracytoplasmic membrane and during bud formation. All catabolic events occur inside the intracytoplasmic compartment of the anammoxosomes, leading to the generation of a proton gradient across the anammoxosome membrane [31,33]. Furthermore, owing to their impermeable membranes, bacteria were shielded from proton diffusion and intermediate toxicity [3]. The catabolic anammox process relied on the activity of five key enzymes: nitrite oxidoreductase, hydroxylamine oxidase, hydrazine dehydrogenase, nitrite reductase, and hydrazine synthase [1]. Figure 2 shows details of the nitrogen cycle, chemical compounds, and enzymes involved. The initial phase relies on nitrate reductase to convert nitrite into nitric oxide. Subsequently, hydrazine hydrolase combines ammonium with nitric oxide to generate hydrazine. Hydrazine dehydrogenase is used to oxidize hydrazine to dinitrogen gas in the final step [37]. The overall stoichiometric equation for the anammox process is shown in Figure 3 [1]. Identifying and characterizing these unique features have played a critical role in advancing our understanding of anammox processes. Figure 3 outlines the various processes of anammox technology used for industrial wastewater treatment: (a) overall stoichiometric equation of the anammox process, (b) completely autotrophic nitrogen removal over nitrite (CANON), (c) oxygen-limited autotrophic nitrification and denitrification (OLAND), and (d) aerobic de-ammonification (DEMON).

## 3. Importance of Anammox Processes

The water treatment process is classified into two main applications—side-stream and mainstream—depending on the presence of microbiota and influent sources [27]. The mainstream method comprises two sequential steps. In the first step, AOB performs partial nitritation (PN) by converting ammonium into nitrite. In the second step, the anaerobic anammox (A) converts the remaining ammonia and nitrite directly into dinitrogen gas [33]. However, partial nitrification/anammox (PN/A) is used as a substitute for traditional approaches. It offers environmental benefits such as a 95% reduction in biodegradable carbon, a 60% decrease in oxygen demand, and 80% sludge elimination: some of the primary objectives of present-day wastewater treatment. Nitritation/anammox-based processes in anammox wastewater treatment methods are more effective and energy-saving in terms of nitrite production [31,38].

A plethora of anammox-based techniques have been explored, including both one- and two-stage systems. These systems have been proven effective for treating various wastewater types, including pharmaceuticals, landfill leachate, piggery, monosodium glutamate, semiconductors, and sludge digester liquids. Numerous nitritation/anammox-based methods have been studied, particularly one-stage and two-stage systems [1]. The single reactor for the high-activity ammonia removal over nitrite (SHARON)-anammox process comprises a two-stage system. Alternatively, the one-stage system comprises processes including single-stage nitrogen removal using anammox and partial nitritation (SNAD), CANON, DEMON, OLAND, denitrifying ammonium oxidation (DEAMOX) [39], and other similar methods that integrate partial nitrification and anaerobic ammonium oxidation (PN/A) [29]. The following section provides a detailed explanation of these processes.

### 3.1. SHARON

**Figure 4:**
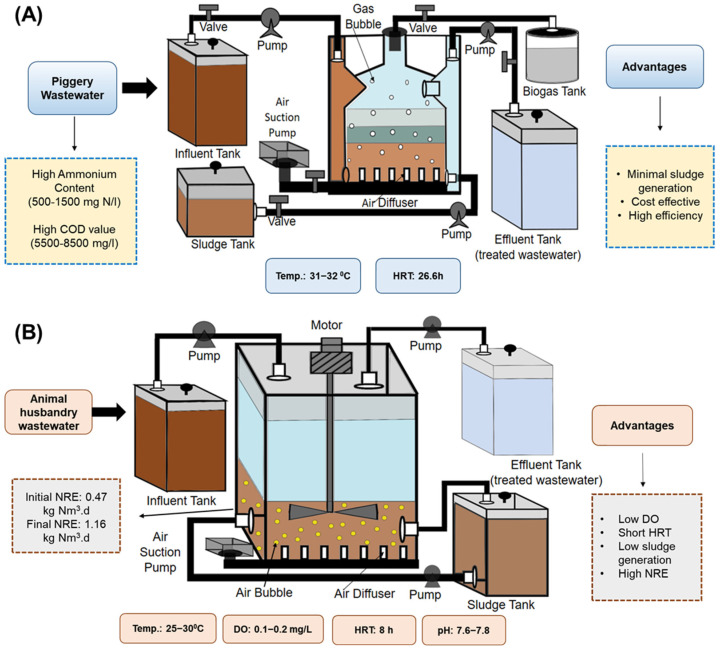
(**A**) Up-flow anaerobic sludge blanket reactor (UASB), (**B**) continuous stirred tank reactor (CSTR).

Piggery wastewater is a significant concern because of its high ammonium content and chemical oxygen demand (COD) value [40]. To address this issue, a stable and efficient laboratory-scale coupled system called SHARON, which utilizes anammox in an up-flow anaerobic sludge blanket reactor (UASB), as shown in Figure 4A, was developed for piggery wastewater treatment. The influent was outsourced from an animal husbandry facility containing NH_4_^+^–N contents ranging between 500 and 1500 mg N/L and COD in the range of 5500–8500 mg/L, located in Changping District, Beijing, China [41]. The anammox reactor used in this study was made of Plexiglass (polymethyl methacrylate) with a height, diameter, and working volume of 1400 mm, 140 mm, and 13.3 L, respectively. The activated sludge inoculum was procured from the aeration chamber of the facility responsible for wastewater treatment. A temperature of 31–32 °C and a hydraulic retention time (HRT) of 26.6 h were consistently upheld to maintain optimal conditions. After 38 days, the nitrogen removal rate reported was 1.3 mg N/L, which increased to 90.4 mg N/L after 55 days and eventually stabilized after 228 days with a value of 420 mg N/L [41]. The SHARON system offers several potential benefits, such as cost-effectiveness, high efficiency (up to 90% ammonia removal), energy efficiency, environmentally friendly operation, and minimal sludge generation. Figure 4A illustrates UASB.

### 3.2. CANON

CANON integrates the anammox process with partial nitrification within a solitary chamber of a reactor that accommodates both aerobic AOA and anammox microbiota [42]. The stoichiometry of the CANON process is shown in Figure 3b [43]. Swine farm biogas digesters are typically high in organic contaminants and nutrient levels, with NH_4_N levels averaging 259 mg/L, COD levels at 463 mg/L, and a combined organic and inorganic nitrogen content of 536 mg/L. To meet the wastewater effluent parameters of livestock farms (total nitrogen content below 150 mg/L and COD below 300 mg/L), these effluents must be treated before discharge. A continuously stirred tank reactor (CANON-CSTR) is a popular option for this purpose owing to its easy installation, operation, and maintenance, as well as reduced setup costs and smaller footprint [44]. Third et al. [45] demonstrated that the CANON process primarily relies on the beneficial interaction between Planctomycete-like anaerobic and Nitrosomonas-like aerobic ammonium-oxidizing microbes.

A laboratory-scale CANON-CSTR reactor, shown in Figure 4B, was used to treat anaerobically pre-treated husbandry wastewater collected from swine and cattle farms in the copper Cu Chi district, Vietnam. The reactor was made of a stainless-steel cylinder with a working volume, diameter, and height of 38.9 L, 300 mm, and 700 mm, respectively. The CANON reactor was seeded with aerobic AOB by introducing landfill leachate pre-treated inside a partial nitrification sequencing batch reactor (PN-SBR). In contrast, an in-use internal circulation anammox reactor containing mid-treated municipal landfill leachate was used as an inoculum for anammox seeding. Granular aerobic sludge collected from winery wastewater treated in a UASB was added during the enrichment phase. The reactor operated for 80 days using wastewater from animal husbandry units that underwent anoxic treatment and then with synthetic wastewater for 106 days. During the enrichment phase, the temperature was maintained at 25–30 °C, DO levels were set to 0.1–0.2 mg/L, and pH was optimized to 7.6–7.8 to attain favorable enrichment outcomes. The mean values of the nitrogen loading rate (NLR) rose incrementally, first to 0.95 kg Nm^3^/d and then to 1.16 kg Nm^3^/d, following an initial starting point of 0.47 kg Nm^3^/d, leading to a decrease in HRTs from 16 to 8 h, and then to 6 h. The nitrogen removal rate achieved its highest consistency and level during the operational phase when the HRT was set to 8 h, and the NLR was maintained at 0.95 kg Nm^3^/d. The CANON-CSTR process offers several advantages, including the use of a simple and inexpensive biocarrier, low DO levels (<0.2 mg/L), short HRT (8 h), low sludge generation, high nitrogen removal efficiency (NRE; 78%), and no outsourced carbon requirement. Therefore, it was a valuable approach for wastewater treatment [44]. Using a fixed-bed reactor (FBR) and adding a mixture of partial nitrification and anammox sludge quickly initiated the CANON process. An operation period of 94 d yielded a total nitrogen (TN) removal efficiency of 91.8%, and the highest gross TN removal quantity recorded was 184 g m^3^/d. The CANON process is robust against loading shocks and recovers from an unstable state. The mature biofilm in the reactor had a porous and microporous structure with an aggregated shape and was dominated by anammox and AOB microbial populations [46]. Figure 4B shows the details of the CSTR.

### 3.3. OLAND

The OLAND system is used for nitrogen elimination from nitrogen-rich wastewater, utilizing a single-step process in which regular nitrifying sludge serves as a biocatalyst in the reaction [47]. SBRS are commonly used in OLAND applications because of their operational flexibility. These reactors allow for simple changes in the volume of effluent withdrawn from the total reactor volume in each cycle and changes in the aerobic/anoxic reaction time, cycle duration, feeding regime, and minimum terminal velocity required for aggregates to settle [48]. The stoichiometry of the OLAND process is mentioned in Figure 3c [47]. When implementing the OLAND technique on a laboratory scale using a rotating biological contractor (RBC) reactor under high-salinity conditions (30 g/L), an 80% reduction in nitrogen was achieved by applying an NLR of 0.73 g N/L/D [27]. However, the OLAND process had several limitations. Firstly, the anammox microorganisms grew slowly in the granular medium, flocs, and biofilm, leading to a prolonged start-up period. Secondly, the activity of ammonia-oxidizing bacteria must be higher than that of anammox to accumulate nitrite nitrogen, upon which nitrogen removal depends. Finally, to achieve the maximum nitrogen elimination efficiency, limited nitrate nitrogen generation is required [27].

### 3.4. DEMON

**Figure 5:**
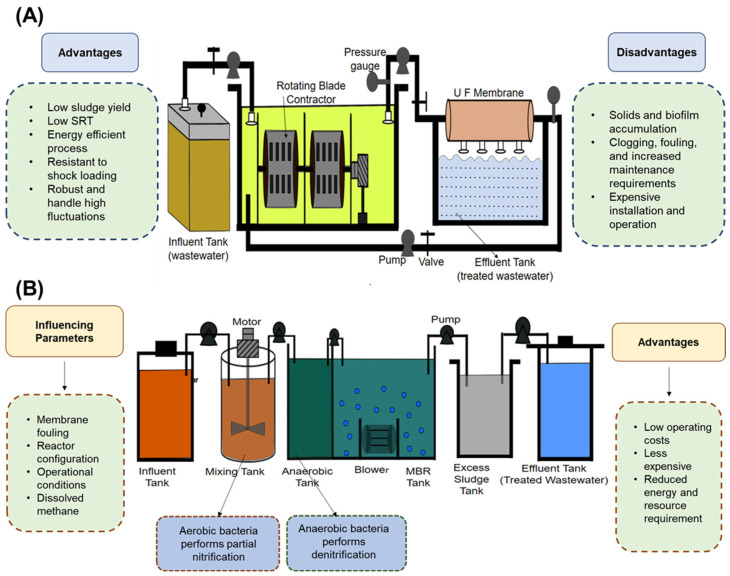
(**A**) Rotating biological contractor (RBC) bioreactor, (**B**) membrane bioreactor (MBR).

The DEMON process is a novel technique first discovered in an RBC, as shown in Figure 5A, which was fed with ammonia-rich landfill leachate and exhibited an NRE of 90% [49]. Ruhrverband constructed a de-ammonification system using the SBR method at the Plettenberg WWTP in 2007, removing up to 95% NH_4_^+^-N and more than 85% total nitrogen [50]. The stoichiometry of the DEMON process is illustrated in Figure 3d [49]. Figure 5A summarizes the details of the RBC. The Marselisborg WWTP in Aarhus, Denmark applied the mainstream DEAMOX method for approximately three years. The reactor’s nitrification/denitrification chambers were fed waste-activated sludge (WAS) procured from a side-stream DEMON bioreactor. The study revealed an 800–900-fold decrease in anammox conversion rates compared to those observed in the side-stream DEMON. Furthermore, only 1% gross nitrogen elimination was achieved in the mainstream reactor. In addition, the anammox bacterial population in the nitrification/denitrification tank was insufficient to have a considerable impact and had no tolerance to colder temperatures [51].

Marie et al. [52] reported that the DEMON method for side-stream processing of ammonia-rich wastewater is efficient and effective. However, several challenges must be addressed before the mainstream adoption of this method can occur. These include poor anaerobic ammonia-oxidizing bacterial retention, insufficient nitrite-oxidizing bacterial suppression, and the regulation of nitrite and soluble COD amount [53]. The authors reported on a full-scale municipal wastewater treatment plant in Austria, where side-stream DEMON^®^ biomass was consistently removed over 1.5 years. The retention and enrichment of granular anammox biomass were regulated using a hydrocyclone and monitored. During an 8-month application span, ammonia and nitrite underwent anoxic-coupled depletion for the first time under ex situ conditions. An increased nitrite formation rate (68% of the total NOx-N) was observed compared to nitrate under oxygenic conditions. The operational plan did not adversely affect the side-stream PN/A performance, and the nitrogen removal rate doubled during the second half of the mainstream monitoring process. The advantages of this method include cost-effectiveness, reduced oxygen consumption in mainstream wastewater treatment plants, and reduced wastewater discharge fees [52]. In conclusion, forming appropriate anammox bacteria (AnAOB) during operation of the mainstream system requires a combination of constant sidestream-DEMON^®^ biomass transfer and granular solid retention time (SRT) enhancement using a hydrocyclone. The findings of this study offer insights for addressing the challenges of the mainstream adoption of the DEMON method [52,53].

### 3.5. DEAMOX

DEAMOX is an effective method of treating wastewater containing both ammonia and nitrate. In this process, sulfide is used as a reductant to reduce nitrate to nitrogen. The DEAMOX process is particularly suitable for the sulfurization of nitrogen pollutant-based wastewater [54]. An innovative DEAMOX process has been designed using attached sponges as carriers in a sequencing biofilm batch reactor named SBBR [55]. After 240 days of operation, the overall NRE remained at 93.0%. The amount of protein-like substances and tightly bound extracellular polymeric substances within the biofilm steadily increased throughout the experiment. The final measurements recorded values of 180 and 142 mg-Ng volatile suspended solids (VSS)/L, respectively, notably higher than the initial values of 65.6 and 46.1 NgVSS/L/h. Furthermore, the highest anammox working inside the biofilm revealed appropriate NH_4_^+^-N elimination rates of more than 4.29 mg-Ng VSS/L/h compared with those of suspended sludge (2.56 mg-Ng VSS/L/h). Anammox bacteria were found in more significant quantities in the biofilm than before the establishment of the biofilm, increasing from 1.9% to 11.5% according to quantitative polymerase chain reaction data [55]. These findings suggest that the carriers developed mature biofilms and that the anammox microbiota remained sufficiently concentrated inside the DEAMOX-SBBR system.

### 3.6. SNAD

Ding et al. [56] developed and applied an SBR for SNAD to treat domestic sewage. This study used suspended-activated sludge instead of biofilm- or granule-activated sludge. During the baseline period of wastewater treatment with high ammonium content, maximum nitrogen and ammonia elimination rates were attained. In the experimental phase, the SNAD process achieved 86.1% gross nitrogen elimination, and only 1.02 mg/L of nitrate concentration remained in the effluent. Anammox (*C. brocadia)* and denitrification (*C. thauera*) contributed 89 and 11% of the total nitrogen removal, respectively. These findings suggest that SNAD using suspended activated sludge can effectively treat domestic sewage.

Aquaculture wastewater was effectively treated using SNAD without additional organic carbon. Lu et al. [57] operated a bioreactor for 180 days, and it attained stability after 60 days with mean values of 0.47 mg/L, 0.26 mg/L, 0.75 mg/L, and 0.27 mg/L for nitrite, ammonia, nitrate, and COD, respectively, in the treated effluent. *Pseudoxanthomonas* was the predominant genus in the biofilm samples. Under high DO conditions, the SNAD bioreactor effectively removed COD and nitrogen from the recirculating aquaculture system. These results demonstrate the potential of the SNAD process for treating aquaculture wastewater without adding organic carbon.

The SNAD process is a promising wastewater treatment technology that requires two key factors for optimal performance: a prolonged SRT and precise control of carbon-to-nitrogen (C/N) and DO concentrations. Prolonged SRT ensures that the anammox bacteria are active in low ammonium-nitrogen conditions and facilitates the coexistence of diverse functional bacteria to establish a robust microbial community that drives the desired biotransformation [58]. Researchers have explored various approaches to extend SRT, such as using absorbent gravel in artificial wetlands, which has been demonstrated as a steady coupling process with up to 91% TN elimination efficiency. Additionally, novel biofilm carrier materials, such as corncobs, have been employed to achieve up to 92.5% TN elimination efficiency [59]. Overall, these findings highlight the critical role of the SRT and optimal C/N and DO concentrations in achieving effective SNAD performance.

### 3.7. Partial Single-Reactor System for High-Activity Ammonium Removal over Nitrite

The partial single-reactor system for high-activity ammonium removal over nitrite (pSHARON/A), also referred to as PN/A technology, is an innovative two-reactor autotrophic denitrification system that relies on the activity of the anammox microbiota to achieve biological autotrophic nitrogen removal. In the first reactor, AOB and AnAOB work in tandem, performing PN to convert half of the NH_4_-N into NO_2_-N. The effluent from the PN tank is transferred to the second anammox tank, where the AnAOB perform denitrification at an increased rate, as shown in Figure 5B [27].

Compared with conventional nitrogen removal systems, PN/A technology is less expensive, and operating costs can be reduced by up to 90% [27,60]. Moreover, recently, the pSHARON/A system was found to be more effective than other autotrophic technologies because it significantly reduced the amount of energy and resources required to treat NH_4_N-rich wastewater [27]. These advantages make PN/A technology a promising approach for nitrogen removal in wastewater treatment, with the potential for broader applications in the future. Figure 5B provides the details of the membrane bioreactor.

### 3.8. Integrated Fixed-Film Activated Sludge

**Figure 6:**
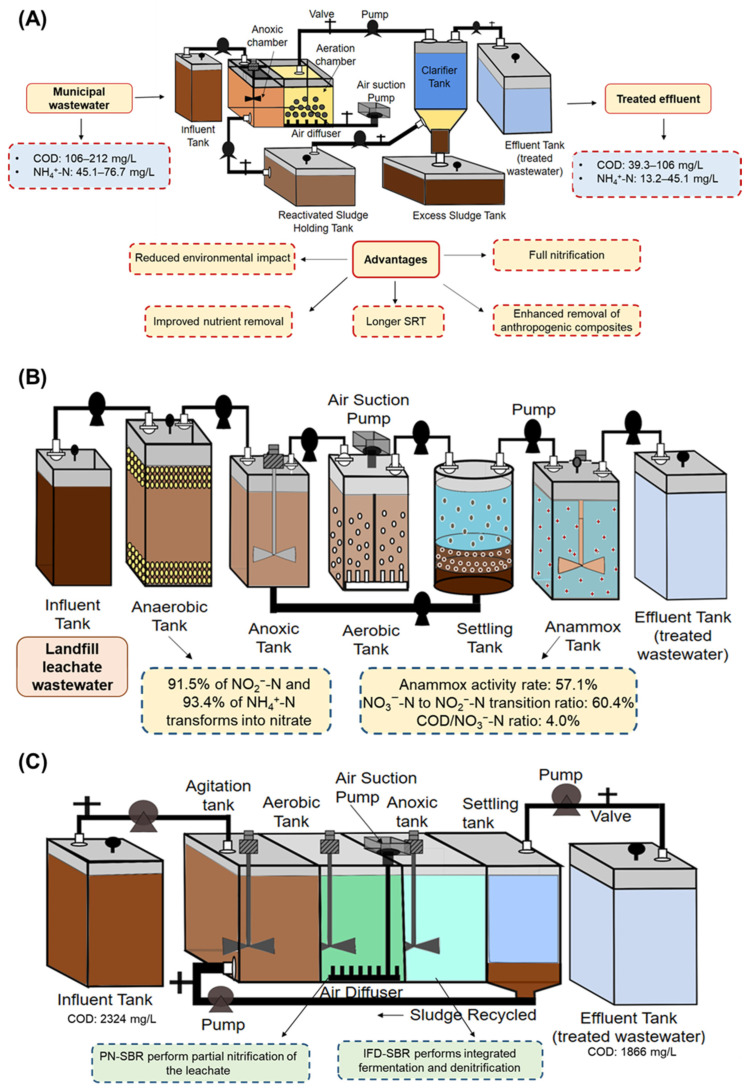
(**A**) Integrated fixed film activated sludge (IFAS) bioreactor, (**B**) landfill bioreactor (LFBR), (**C**) sequencing batch reactor (SBR).

The integrated fixed-film activated sludge (IFAS) method is a sustainable wastewater treatment approach that combines attached and suspended growth systems to improve a moving-bed biofilm reactor [61]. To enhance the treatment of high ammonia-containing municipal wastewater, a SNAD-IFAS bioreactor was constructed by inoculating nitritation-suspended sludge, as shown in Figure 6A [62]. The system exhibited high COD and gross nitrogen removal efficiencies, with 39.3 and 13.2 mg/L output values. Notably, this study found that AOB self-generated within the SNAD-IFAS system. These bacteria could be retained within the system, suggesting potential applications for wastewater treatment facilities.

IFAS offers several advantages over traditional activated sludge methods, including reduced environmental impacts, longer solid retention time, improved nutrient removal, full nitrification, and enhanced removal of anthropogenic composites. Numerous pilot and full-scale studies have shown that IFAS is a desirable choice for wastewater treatment. It can improve sludge settling characteristics, increase operating stability, and achieve a greater than 90% reduction in ammonia and chemical oxygen demand. Ongoing developments in IFAS reactor design include frameworks for methane synthesis, algae-based energy generation, and microbial fuel cells [61]. Figure 6A shows the details of the IFAS bioreactor.

### 3.9. Simultaneous Nitrogen and Phosphorus Removal

In wastewater treatment, the simultaneous removal of phosphorus and nitrogen is a significant obstacle that needs to be addressed. The simultaneous nitrogen and phosphorus removal (SNPR) process is a promising solution that combines intrinsic partial denitrification with denitrifying dephosphatation in SBR [63]. This novel system, which consists of granular sludge anammox and suspended sludge, was designed to treat synthetic sewage-like wastewater. The process, with an average input C/N ratio of 2.9, achieved an impressive level of nitrogen (93.9%) and phosphorus (94.2%) removal when operated over 200 days [15,64]. This study found that the anammox process was responsible for eliminating 82.9% of the total nitrogen, which was attributed to the persistent nitrite synthesis resulting from nitrate’s intrinsic partial denitrification. Most phosphorus removal occurs through denitrifying dephosphatation using nitrate as an oxidant, substantially reducing the need for oxygen and carbon supply. Interestingly, 1.1%, 8.4%, and 1.5% phosphorous-accumulating organisms, anammox bacteria, and glycogen-accumulating organisms were reported to inhabit the system, respectively [64]. These findings indicate that the SNPR process can treat wastewater with high efficiency and low energy consumption. Because of its ability to simultaneously remove nitrogen and phosphorus, it represents a promising strategy for sustainable and eco-friendly wastewater treatment.

### 3.10. PN/A and Partial Denitrification-Anammox (Three Stage System-Based Processes)

Wang et al. [65] presented a novel one-piece flow-coupled method that combined PN/A and partial denitrification-anammox (PD/A) and attained a maximum elimination efficacy of nitrogen from landfill seepage wastewater. In the PN reactor, 91.5% of NO_2_^−^-N and 93.4% of NH_4_^+^-N transformations were achieved. The feed (38%) and nitrite produced by the PN reactor were mixed and then fed into the anammox reactor. After release into the PD/A tank, the nitrate created in the anammox tank was converted into nitrite and eliminated via anammox. The anammox activity rate and the NO_3_^−^-N to NO_2_^−^-N transition ratio in the PD/A reactor were 57.1% and 60.4%, respectively, at a COD/NO_3_^−^-N ratio of 4.0. The effluent had a total TN concentration of 15.7 mg/L, while TN was eliminated with 98.8% efficiency. By integrating PN/A and PD/A and introducing an outsourced carbon (COD/NH^4+−^N) ratio of 0.28, more nitrogen could be eliminated from the landfill leachate. The most abundant groups in the PD/A and anammox bioreactors were *C. kuenenia* and *C. brocadia*, respectively [65]. Overall, this study suggests that the one-piece flow coupled method using a landfill bioreactor, as shown in Figure 6B, is a potentially effective approach for eliminating the nitrogen present in landfill leachate wastewater. Figure 6B shows the details of the landfill bioreactor.

### 3.11. Simultaneous Ammonium Oxidation Denitrification

Zhang et al. developed a novel simultaneous ammonium oxidation denitrification (SAD) method for treating stabilized landfill leachate that contains more ammonia contaminants and few organic materials [66]. The SAD method comprises three stages: PN-SBR, integrated fermentation and de-nitrification (IFD-SBR) using concentrated external WAS and an anaerobic bioreactor, and anammox in the SAD-UASB to eliminate the remaining nitrite and ammonia produced during fermentation. Prolonged operation for 280 days showed good removal rates for TN of 98.3%, output with a TN concentration of 16.7 mg/L, and external sludge removal levels of 2.5 kg/m^3^ per day. The nitrogen removal rates for the SAD-UASB and IFD-SBR systems were 12.3% and 81.9%, respectively. A favorable interaction was observed between *C. brocadia* (1.5%) and *C. thauera* (PD bacteria) (4.3%) in the SAD-UASB reactor. These findings suggest that SAD is potentially effective for efficiently eliminating the nitrogen-based contaminants present in mature landfill leachate effluents [53,67].

### 3.12. Partial Nitrification and Integrated Fermentation Denitritation

Zhang et al. [66] developed a new technique for enhancing nitrogen elimination from mature landfill leachate with a minimal C/N ratio (1:1) and reducing WAS during 300 days of operation. This novel technique, called partial nitrification and integrated fermentation denitritation (PNIFD), utilized two SBRs, as shown in Figure 6C. The primary reactor, an aerobic reactor (PN-SBR), was used for partial nitrification of the leachate, whereas the secondary reactor, an anoxic bioreactor (IFD-SBR), performed PN-SBR effluent and WAS-integrated fermentation and denitrification. The results showed a 95.0% efficiency at a rate of 0.63 kg/m^3^ per day in eliminating nitrogen at the end of the operational period. Moreover, after complete processing, the influent (mature landfill leachate) with a COD of 2324 mg/L showed a COD reduction efficiency of 19.7%, leaving only 1866 mg/L of COD in the effluent. Throughout the IFD-SBR operational cycle, approximately 53.7% of the entire external sludge was eliminated, with a decrement rate of 5.1 kg/m^3^ day. The PNIFD technique offers a simple and effective solution for simultaneously eliminating nitrogen and reducing WAS in mature landfill leachates. The study findings indicate that the PNIFD technique is a promising approach for eliminating nitrogen from leachate with a low C/N ratio. Figure 6B provides the details of the SBR.

## 4. Factors Affecting Anammox Process

### 4.1. Instability in Interaction Between Functional Microbial Communities Involved in Anammox

Anammox is a crucial biological process used to eliminate nitrogen from wastewater. This process involves the interaction of several microorganisms including AnAOB, AOB, heterotrophic bacteria (HB), and NOB [37]. AnAOB is the most critical microorganism in the process and relies on a constant and stable supply of both ammonia and nitrite to operate effectively [68]. Although ammonia is readily available in untreated wastewater, nitrite levels are usually low and must be generated through nitrification. During nitrification, AOB utilizes ammonia monooxygenase to convert ammonia into nitrite. However, NOB readily converts nitrite into nitrate, which is challenging for anammox.

HB is also essential for this process, as it generates nitrite as an intermediate during full denitrification. Enzymes such as nitric oxide reductase, nitrate reductase, nitrous oxide reductase, and nitrite reductase are involved in denitrification. When HB is transformed into nitrite, dinitrogen is generated through biochemical steps. However, this leads to unbalanced nitrite accumulation in the system, which is unfavorable for anammox [37,69].

Microbes compete fiercely for similar substrates in the system. Ammonia serves as a reductant for both AOB and AnAOB. Nitrite serves as an oxidant for AnAOB, a reductant for NOB, and a substrate for the reduction of nitrite by HB. DO serves as an oxidant for both NOB and AOB, whereas HB uses DO to remove organic matter [37]. Therefore, proper monitoring of the interactions between these microorganisms is crucial for maintaining the stability of a single-stage anammox process. Maintaining anammox biomass, ensuring significant AOB activity, and preventing the overgrowth of NOB and HB are crucial to achieving successful nitrogen removal [29].

### 4.2. Sustainability of Maintaining Anammox Biomass (Granular/Biofilm)

Maintaining a stable anammox biomass is crucial for effectively operating a one-stage anammox process because anammox bacteria have a slow growth rate. Anammox microbiota are directly responsible for the efficacy of anammox bioreactors. Recently, two effective methods for maintaining anammox biomass were found to generate compact aggregates of microorganisms (granular sludge) and utilize support media (carrier materials) to create biofilms [70]. Although a biofilm requires the coupling of carriers for its growth, AnAOB have been found to grow unintentionally as biofilms on the walls of some bioreactors [71]. Additionally, biofilms have been created using zeolite particles as a carrier medium, resulting in increased anammox biomass enrichment and decreased biomass washout in the outflow to levels below 3 mg VSS/L [72].

The application of a submerged module made of hollow fibers is an effective strategy for preventing the loss of biomass from a membrane SBR [73]. Furthermore, non-woven membranous bioreactors have been successful in maintaining a significant amount of biomass through aggregate formation within the bioreactor [37]. In both laboratory-scale anammox-SBR and pilot-scale IFAS processes, the leachate was treated using a polyethylene sponge carrier, which has a high efficacy for nitrogen removal [74,75]. The use of this carrier resulted in an increase in anammox gene levels from 1.3% to 13.3% owing to biofilm protection [37]. These findings highlight the importance of utilizing effective methods to sustain anammox biomass in one-stage anammox processes for optimal performance of anammox bioreactors.

Granular sludge is a microbial community that has received considerable attention in recent years owing to its unique characteristics, such as enhanced biomass retention and quicker settling speed. These properties lead to reduced amounts of biomass in the suspension, making it an attractive option for wastewater treatment [63]. The production of anammox granular sludge offers several advantages over conventional sludge treatment methods. One of the most significant advantages is the increased nitrogen elimination rate, which can lead to savings in infrastructure expenditure. The maximum nitrogen elimination rate attained in a lab-scale anammox granular sludge method ranges from 74.3 to 76.7 kgN/(m^3^ d) [76]. However, optimizing the selection of inoculum sludge and increasing nitrogen loading can improve reactor performance, leading to faster and more efficient production of granular sludge [77].

Despite the advantages of anammox granular sludge production, its application remains challenging. One of the most significant challenges is that granular sludge can effortlessly drift in an enhanced loading capacity, leading to the washout of granules and a decrease in reactor performance. This may have arisen from bubbles in the trapped gas and decreased density [78]. One possible strategy to address the floating issue and the negative impact on activity due to particle size is to recover anammox granules from wastewater and fragment them before putting them back into the reactor [79]. Another approach for preventing the flotation of anammox granules is hydrodynamic control. One strategy involves utilizing constantly running gas-automated circulation inside a UASB. This approach reduces the formation of large gas bubbles and promotes the formation of smaller bubbles, thereby reducing the likelihood of granule washout [37]. Also, strong hydraulic loading can increase the shear force, causing a large gas column to split into several smaller gas bubbles. This approach has been shown to prevent granule flotation and improve reactor performance.

### 4.3. Physiochemical Parameters

Recently, anammox has shown great potential for effectively eliminating nitrogen from wastewater, making it a highly promising technology. However, continuous online monitoring is crucial to ensure steady and secure operation and practical viability. Several parameters are commonly monitored online, including temperature, pH, DO, ammonia, nitrate, nitrite, nitrogen loading rate, and carbon sources [29]. These monitoring efforts can potentially enhance the performance and efficiency of anammox reactors, thereby facilitating their widespread adoption in the wastewater treatment industry.

#### 4.3.1. Temperature

Temperature is a crucial parameter that significantly affects the anammox processes. Temperature fluctuations can alter the physical reactions of anammox and the effectiveness of nitrogen removal [33]. When low-nitrogen effluent is employed in various bioreactors, temperature influences nitrification, microbial population composition, and effluent stability [37]. However, anammox microbiota exhibit wide temperature adaptability ranging between 5 and 40 °C in natural environments, which makes them suitable for various wastewater treatment applications [1]. Notably, anammox bacteria thrive and exhibit optimal performance at temperatures between 30 and 40 °C during mainstream processes. Moreover, the side stream anammox process has been suggested to operate optimally at a temperature between 35 and 40 °C [33]. *C. Brocadia sinica* and *C. Brocadia fulgida* are known to exhibit optimal performance at temperatures ranging between 5 and 6 °C [80]. Conversely, *Candidatus Kuenenia stuttgartiensis* has been observed to thrive in a constant reactor system operating at temperatures below 20 °C [81]. However, the anammox process can be optimized in high-latitude regions, where temperatures ranging between 18 and 23 °C were found to promote rapid growth, relatively high anammox activity, and low levels of free nitrous and ammonia [82].

#### 4.3.2. pH

Fluctuations in pH levels can result in the build-up of harmful compounds that impede the performance of anammox microbiota. The optimal pH range for anammox is between 7 and 8, which helps prevent the suppression of anammox due to excessive free ammonia and nitrous acid [83]. Wang et al. [84] found that the optimal pH range for anammox function and development was between 6.8 and 8.3, with a pH of 8.0, exhibiting the highest activity. The pH range between 8 and 8.5 was found to be dominated by two *Candidatus* spp., namely *C. Brocadia anammoxidans* and *C. Kuenenia stuttgartiensis*, while *C. Anammoxoglobus propionicus* and *C. Brocadia anammoxidans* were found to dominate at pH levels between 6.8 and 7.0 [33]. As the anammox mechanism typically causes the pH level to rise, pH control is crucial for maintaining steady bioreactor operation.

#### 4.3.3. DO

AOB are obligate anaerobes that require strict anoxic conditions for optimal growth and metabolic activity. Anammox efficiency is significantly suppressed even at very low levels of DO, such as 2% air saturation [85]. The anammox mechanism is reversibly inhibited at DO concentrations below 1% air saturation, whereas irreversible blockage occurs at DO levels exceeding 18% air saturation [37,86].

When partial nitrification is necessary at high DO concentrations, anammox biomass in the form of granules can be an effective alternative to anammox biofilms [1]. Recent research has demonstrated that 600 mg/L/day of nitrogen is eliminated under experimental DO levels of 1 and 8 mg/L, as was determined using oxygen microelectrode profiling in a granular anammox bioreactor. Conversely, the anammox development and activity were negligible in the anammox biofilm maintained at a DO level of 8 mg/L. Optimal DO levels for inducing high rates of anammox activity are advised to be less than 0.04 mg/L [1]. Therefore, maintaining strict anoxic conditions is crucial for optimal anammox bioreactor performance.

#### 4.3.4. Nitrogen Loading

The nitrogen content strongly influences the anammox process. The nitrogen elimination rate, growth potential, and dominant bacterial species of anammox systems can be significantly affected by variations in nitrogen concentration levels [87]. Anammox activity may decrease because of low ammonium content and high cell density. Conversely, inadequate nitrite and ammonium levels may promote the growth of harmful microorganisms and hinder the anammox process. Anammox growth may be hindered by nitrate and nitrite levels that surpass 0.07 g/L, but nitrite levels of up to 0.04 g/L can encourage specific anammox activities and growth [33].

#### 4.3.5. Carbon Sources

Anammox is an efficient, economical, and eco-friendly approach for removing nitrogen from wastewater influents with low C/N ratios and is widely acknowledged [88]. However, sustaining anammox activity in a membrane-aerated biofilm bioreactor with COD/N levels above two is not possible [89]. In contrast, a conventional co-diffusion biofilm bioreactor with an identical COD/N ratio exhibited a 50% reduction in the nitrogen elimination rate [29]. Furthermore, the nitrogen elimination efficiency remained unaffected in a biofiltration unit (CANON) inhabiting AOB, denitrifiers, and anammox microbiota fed with feed water with an ammonium content and COD of 320 mg/L and 100 mg/L, respectively [90]. However, the same reactor, inhabiting anammox and AOB, significantly reduced nitrogen elimination efficiency when operated without organic carbon.

#### 4.3.6. Substrate

Ammonium is the primary source of nitrogen in most wastewater treatment plants. However, the impact of free ammonia (FA) concentration on the anammox process surpasses that of ammonium. Therefore, FA is considered the primary inhibitor of anammox [29]. Adequate nitrogen levels were achieved on the 116th day, indicating successful initiation of the anammox reactor with a nitrogen content of 0.72 kg N m^3^/d. The reactor exhibited satisfactory performance, as it could withstand free nitrous acid levels of 39.5 g/L and FA levels of 13.6 mg/L, surpassing the endurance of anammox reactors with granular sludge. Anammox inhibition occurs at free nitrous acid and FA concentrations of 77.0 and 29.6 mg/L, respectively [91]. Anaerobic ammoxidation depends on the presence of nitrite; however, this reaction is hindered under excess nitrite content. Although nitrate accumulation is not a primary inhibitor, it can somewhat affect the microbial community balance in the reactor. Excessive aeration in the reactor is the main contributor to nitrite and nitrate accumulation [92], and it can be suppressed by microbial electrochemical-based technologies using *Alcaligenes* [65,93]. The regulation of FA, nitrite, and nitrate content in the anammox system is crucial, particularly the nitrite content during the initial phase of the one-stage system, where AnAOB are outcompeted by AOB, as an increase in nitrite concentration can substantially affect the anoxic microbiota [29].

### 4.4. Other Environmental Factors

#### 4.4.1. Nitrite

The anammox equation provides a theoretical nitrite-to-ammonium ratio of 1.32:1, with nitrite playing a critical role as the primary substrate for anammox microorganisms. However, under anaerobic conditions, the same compounds can be toxic to these microorganisms [33]. The degree of nitrite inhibition varies across the different anammox systems and inoculum sources. Anammox systems operate efficiently at a baseline ammonium (42 mg N/L) concentration but are inhibited at ammonium concentrations exceeding 185 mg N/L. The ability of anammox to transform nitrogen into nitrogen gas continually diminishes when nitrite concentrations exceed threshold values [83]. The term “IC_50_” refers to the nitrite concentration at which the anammox activity is reduced by 50%. The IC_50_ value varies across different inoculation sources, with UASB granular sludge exhibiting a maximum IC_50_ of 240 mg/L, while the IC_50_ values for moving bed biofilm reactor biofilm and SBR sludge were reported to be 85 and 98 mg/L, respectively [94]. However, the effects of nitrite levels on the activity of anammox bacteria cannot be anticipated and requires further experimental investigation. Therefore, regulating the nitrite concentration is essential to maintain anammox system effectiveness by keeping it under a previously defined threshold [83].

#### 4.4.2. Sulfide

The presence of sulfur dioxide in anaerobic wastewater systems under anoxic conditions is often attributed to the degradation of organic compounds and sulfate reduction. Therefore, enhancing our understanding of the harmful impacts of sulfide on the functioning of anammox bacteria is necessary to ensure the stable operation of the anammox process [1]. In a batch experiment, sulfide-S was introduced into the feed water at a rate of 264 mg/L via granular anammox sludge inside serum vials, resulting in a 50% reduction in anammox activity [83]. In another investigation, the anammox inoculum resulted in a 17.2% decline in NRE in a UASB bioreactor under steady feed water conditions containing 40 mg/L of sulfide-S. These results suggest that changing the operating mode and reactor can significantly alter the impact of sulfides on the same anammox population [33].

#### 4.4.3. Toxic Metals

The presence of hazardous metals in wastewater treatment facilities is a pressing concern, with major contributors including landfill leachate and industrial wastewater [1]. These toxic metals have been reported to cause anammox cell death or inhibition, with lead, copper, nickel, chromium, manganese (Mn), zinc, and cadmium being examples of commonly identified hazardous metals in anammox biota [33]. Heavy metals present in concentrations ranging between 19.3 and 176 mg/L can result in a 50% reduction in anammox activity [95]. Of these metals, Mn^2+^ and copper have been identified as the most and least inhibitory, respectively, concerning anammox function. Therefore, controlling the concentrations of these heavy metals within an optimum range is essential to maintain the effectiveness of the anammox process.

#### 4.4.4. Toxic Organic Compounds

Studies have examined the impact of alcohol on anammox activity because anaerobic sludge may undergo alcohol fermentation from organic materials during the early stages of the enrichment process, potentially hindering anammox activity [96]. Methanol has been found to completely suppress the anammox process in marine sediments at concentrations within the range of 96–128 mg/L, which is attributed to stimulation of the denitrification process at these concentrations. However, the successful enrichment of methanol-resistant anammox bacteria, specifically *C. brocadia*, has been achieved using methanogenic sludge [33].

#### 4.4.5. Antibiotics

Antibiotics have become important tools for treating infections in humans, animals, and aquaculture. Moreover, they are frequently used as prophylactic measures on breeding farms and in aquaculture. However, owing to their widespread use and dissemination, antibiotics have become pervasive in aquatic ecosystems, manifesting in various concentrations [97,98]. The antibiotics commonly found in aquatic systems include penicillin, ampicillin, sulfamethazine, chloramphenicol, amoxicillin, tetracycline, sulfathiazole, florfenicol, oxytetracycline, and oxytetracycline [23]. Within an anammox system, higher doses of antibiotics reduce nitrogen removal. Penicillin dosages of 1 and 100 mg/L suppress anammox effectiveness by 17% and 36%, respectively [97]. Similarly, at a dose of 800 mg/L of ampicillin or 200 mg/L of chloramphenicol, anammox effectiveness was significantly reduced by more than 90%. In the first three days following exposure, anammox activity was reduced by 98% with 200 g/L of chloramphenicol, although acclimatization led to a marginal increase in anammox activity after three days [23,33].

## 5. Anammox Bacterial Diversity and Processes Efficiency

Anammox technologies offer promising perspectives for treating ammonium-rich wastewater. Table 1 summarizes the different anammox bacteria identified to date and their biophysical properties [99,100,101,102,103,104,105,106,107,108,109]. One of the principal benefits of anammox technologies is their ability to efficiently treat NH_4_-containing wastewater, especially under minimal biological availability of degradable carbon [1,15]. These technologies also have the potential to substantially lower operational costs and reduce the volume obligations for wastewater treatment facilities. Table 2 elaborates on the engineering aspects of the one-, two-, and three-stage anammox processes [110,111,112,113,114]. Additionally, anammox technologies have attracted interest due to their environmentally friendly nature [30,34,111]. A comparison between the most-used bioreactors in the anammox process and the controlled parameters and resulting NRE is briefly presented in Table 3 [115,116,117,118,119,120,121,122,123].

## 6. Prospects of Anammox Process

As a clean technology, anammox offers promising perspectives for treating ammonium-rich wastewater. One of the principal benefits of anammox technologies is their ability to efficiently treat NH_4_-containing wastewater, especially under minimal biological availability of degradable carbon [1,15]. These technologies also have the potential to substantially lower operational costs and reduce the volume obligations for wastewater treatment facilities. Additionally, anammox technologies have attracted interest due to their environmentally friendly nature [30,34,111]. Implementing the anammox process for treating industrial wastewater remains challenging owing to the prolonged start-up cycle, slow growth rate with a doubling time of 10–12 days, and low cell output [91,119]. Ongoing research is exploring different seeding sludges to enrich anammox. However, maintaining anammox and AOB’s activity and growth rates is challenging because of the organic content, temperature, minimal feed water ammonia content, and other parameters. PD could be considered a more reliable and consistent source of nitrite, and future research should focus on its experimental utility in combination with PN/A, PD/A, and other processes [114,115,123]. Additionally, the development and implementation of more efficient three-stage systems are recommended. Operating conditions and ecological stressors should be monitored and regulated to ensure optimal anammox performance. Hybrid system processes utilizing flocs, granules, or biofilm carriers may enhance nitrogen removal efficiency [4,6,12,21,28]. In addition, new carrier substances or composite carriers that promote microbial attraction and adsorption should be explored. Overall, managing wastewater through the biorefinery concept by integrating biological and bioelectrochemical systems for efficient nutrient recovery can be desirable for sustainable development [124,125].

## 7. Conclusions

The removal of nitrogen compounds from industrial wastewater containing high concentrations of ammonium, nitrite, and nitrate is paramount. Anammox has emerged as a more efficient and effective approach than traditional methods. The selection of mainstream and side-stream processes is critical for coupling different bioreactors to achieve optimal results and additional benefits. Numerous one-, two-, and three-stage-based systems have been developed to maximize the efficiency process. However, operational (such as the interaction between functional microbial communities, maintaining anammox biomass, temperature, pH, DO, nitrogen loading, carbon sources, and substrate), and environmental inhibitory factors (such as toxic metals, nitrite-to-ammonium ratio, toxic organic compounds, and antibiotics) pose a substantial challenge requiring close monitoring and regulation to ensure optimal performance. Future research should focus on seed enrichment and using different carriers, such as granular sludge, flocs, and biofilms, to retain biomass. These research areas are crucial for improving the efficacy of anammox processes and ensuring successful industrial wastewater treatment via clean technology.

## Figures and Tables

**Table 1:** Different anammox bacteria identified to date and their different biophysical properties (sources, nutritional form, optimum regulatory temperature, and pH).

Microorganism	Source	Form of Nutrition	Physiological Conditions		Reference
			Temperature (°C)	pH	
*Anammoximicrobium moscowii*	Wetland/wastewater sludge	Chemolithoautotrophic	19–22	7.8–8.3	[99,100]
*Anammoxoglobus propionicus*	Municipal synthetic wastewater	Chemolithoautotrophic/propionate oxidizing	33	7.0–7.3	[63,101,102]
*Brocadia anammoxidans*	Landfill leachate wastewater	Chemolithoautotrophic	20–43	6.7–8.3	[19,103]
*Jettenia asiatica*	Swine wastewater	Chemolithoautotrophic	30–35	8.0–8.5	[104,105]
*Kuenenia stuttgartiensis*	Municipal wastewater/freshwater	Chemolithoautotrophic	25–37	6.5–9.0	[106,107]
*Scalindua wagneri*	Saline wastewater/marine	Chemolithoautotrophic	22–25	7.0–8.0	[108,109]

**Table 2:** Engineering aspects of various one-, two-, and three-stage anammox processes.

Stages	Process	Treated Object	Bioreactor (Working Volume)	Seed Sludge	Influent Parameters (Feed)	Effluent Parameters (Treated Object)	NRE (%) [AC] (%)	Reference
**One**	DEAMOX	Synthetic wastewater	SBBR (10 L)	DEAMOX suspended sludge	NH_4_^+^-N: 151–157 mg N/L NO_3_^−^-N: 166–201 mg N/L HRT: 5–10 h Temp: 30 ± 5 °C	NH_4_^+^-N: 0.68 mg N/L NO_3_^−^-N: 1.92 mg N/L HRT: 5–10 h Temp: 30 °C	>90	[55]
	CANON	Synthetic wastewater	FBR (2.6 L)	- ^a^	NH_4_^+^-N: 50 mg N/L Temp: 32 °C DO: <0.3 mg/L HRT: 1 d pH: 7.8–8.0	Temp: 32 °C DO: <0.3 mg/L HRT: 1 d pH: 7.8–8.0	91.8	[46]
	SNAD	Municipal wastewater	IFAS (8 L)	Lab-scale SBR	NH_4_^+^-N: 45.1 mg N/L NO_2_^−^-N: <0.3 mg N/L COD: 106–212 mg/L Temp: 18.5–30 ± 1 °C C/N ratio: < 3	TN: 13.2 mg N/L COD: 39.3 mg/L ORE: 78.8%	92.8	[62]
**Two**	SHARON/A	Ammonium-rich wastewater	Control LFBR (43 L)	-	-	TN removal efficiency: 84%	71.0	[113]
			SHARON/A LFBR					
	SPND/A	Domestic wastewater	SPND-SBBR (10 L)	Nitrification sludge	NH_4_^+^-N: 71.4 mg N/L NO_2_^−^-N: 0.2 mg N/L NO_3_^−^-N: 0.3 mg N/L COD: 239 mg/L C/N ratio: 3.4 pH: 7.1–7.5 HRT: 2 h DO: 0.1 mg/L	NH_4_^+^-N: 0.6 mg N/L NO_2_^−^-N: 0.2 mg N/L NO_3_^−^-N: 7.6 mg N/L COD: 33.2 mg/L	88.2 [75.0]	[114,116]
			A-UASB (2 L)	Anaerobic digestive sludge				
	PD/A	Municipal sewage	PD-SBR (5 L)	-	NH_4_^+^-N: 58.3 mg N/L NO_3_^−^-N: 107 mg N/L COD: 194 mg/L HRT: 3.1–3.6 h Temp: 14.8–28.2 °C	TN: 11.0 mg N/L C/N ratio: 1.7	92.8 [78.9]	[110,111]
			A-UASB (3.2 L)					
	SNPR	Synthetic sewage wastewater	SNPR-SBR (10 L)	PD sludge from EPD reactor	HRT: 6.7 h pH: 8.0–8.4 Temp: 27 ± 2 °C NH_4_^+^-N: 60 mg N/L COD: 180 mg/L	NH_4_^+^-N: 2.1 mg N/L TN: 3.6 mg N/L PO_4_^3−^-P: 0.3 mg P/L	93.9 PRE: 94.2 [82.9]	[15,64]
			N-SBR (10 L)	Suspended sludge	DO: <5 mg/L HRT: 5 h pH: 7.3–8.1			
	PN-SAD	Municipal wastewater	PN-SBR (10 L)	PN reactor	NH_4_^+^-N: 52.1 mg N/L NO_2_^−^-N: 0.10 mg N/L COD: 164 mg/L HRT: 6 h Temp: 18.5–28.2 °C DO: <1 mg/L	NH_4_^+^-N: 0.4 mg N/L NO_2_^−^-N: 1.6 mg N/L COD: 38.4 mg/L HRT: 6 h Temp: 18.5–28.2 °C DO: < 1mg/L	97.1 [80.0]	[112]
			SAD-UASB (4 L)	PN reactor				
	DNS/A	Domestic wastewater	PNA-SBR (10 L)	Synthetic ammonia-rich wastewater	NH_4_^+^-N: 300 mg N/L HRT: 20–26 h DO: 0.2–0.8 mg/L Temp: 26.8–13.0 °C	TN: 1.2 mg N/L HRT: 4.5–7.5 h DO: 0.0–0.1 mg/L	99.6 [94.4]	[110]
			PDA-SBR (6 L)					
**Three**	PN/A+ PD/A	Landfill leachate	A/O-CFR (10.5 L)	PN-sludge domestic wastewater	-	ATR: 93.4% NAR: 91.5% FA: 43.5 mg/L FNA: 0.18 mg/L	7.0	[65,93]
			PD/A-UASB (3.5 L)	PD/A SBR reactor	-	TN: 15.7 mg N/L	18.0	
			A/UASB (10 L)	Synthetic wastewater	-	NH_4_^+^-N: 19.6 mg N/L NO_2_^−^-N: 11.5 mg N/L	73.0	
	PNIFD	Mature landfill leachate	PN-SBR	-	COD: 2324 mg/L	NRR: 0.63 kg/m^3^.d COD: 1866 mg/L SRR: 5.9 kg/m^3^.d	95.0 ORE: 19.7	[15,66]
	SAD	Mature landfill leachate	PN-SBR (10 L)	PN-SBR sewage wastewater	NH_4_^+^-N: 1760 mg N/L NO_2_^−^-N: 3 mg N/L NO_3_^−^-N: 4 mg N/L COD: 217 mg/L pH: 7.8	TN: 16.7 mg/L SRR: 2.5 kg/m^3^.d	98.3 [83]	[53,67]
			IFD-SBR (6 L)	PN-SBR sewage wastewater				
			SAD-UASB (2 L)	Granular anammox-PD reactor				

^a^ Not available.

**Table 3:** Comparison between the most-used bioreactors in the anammox process, along with controlled parameters and resulting nitrogen removal efficiency (%).

Types	Feed	IAC (mg N/L)	ICOD (mg N/L)	C/N Ratio	HRT (H)	SRT (D)	pH	Temp. (°C)	DO (mg N/L)	EAC (mg N/L)	NRR (mg N/L.d)	NRE (%)	MO	Reference
MBBR	Municipal wastewater	23.0	54.0	-	11.0	6.80–24.5	7.40	15.0	1.20–0.17	1.90	80.0	-	*Thauera*, anammox bacteria	[115]
SBR	Reject water	220	<180	10/1	2.00–3.00	NA	NA	30.0	-	40–70	80.0 kgN/L.d	80.0	NA	[120]
	Sewage wastewater	33.0–76.9	39.1–184	1.20–2.50	218	-	7.49–7.74	30.0	1.5–3.0	-	94.7	84.0–95.0	*Thauera*, anammox bacteria	[119]
	Low-nitrogen-containing wastewater	6.20	- ^a^	-	5.42	-	-	30.0	-	1.90	0.54 kg N/L.d	86.5	*Thauera*, anammox bacteria	[116]
	Municipal wastewater	65.0	300	-	-	4.60	7.50	25.0	2.50	<5.00	-	90.0	*Candidatus brocadi*	[118]
	Mixed activated sludge	64.0	-	-	-	-	-	28.0	2.00–3.00	2.70	214 g N/L.d	93.0–99.0	*Candidatus kuenenia*	[117]
SBR-IFAS	Municipal wastewater	120	-	<3.20	8.00	25.0	-	30.0	0.40	-	105	90.1	*Candidatus brocadia*, *Candidatus competibacter*	[121]
UASB	Chicken digestate wastewater	330	2868	-	-	-	8.0–8.2	-	-	-	-	57.0	-	[122]
	Synthetic wastewater	100–180	-	-	4.76–1.06	~40.0	6.90–7.20	30.0	-	-	1577 kgN/L.d	93.7	*Candidatus kuenenia, Candidatus brocadia*	[123]

^a^ Not available.
